# The Prospect of Lactoferrin Use as Adjunctive Agent in Management of SARS-CoV-2 Patients: A Randomized Pilot Study

**DOI:** 10.3390/medicina57080842

**Published:** 2021-08-19

**Authors:** Fahad Dhafer Algahtani, Mohamed Tharwat Elabbasy, Mai A. Samak, Adeniyi A. Adeboye, Rafeek A. Yusuf, Mohamed E. Ghoniem

**Affiliations:** 1 Department of Public Health, College of Public Health and Health Informatics, University of Ha’il, Ha’il 2440, Saudi Arabia; dr.algahtani@gmail.com (F.D.A.); tharwat330@gmail.com (M.T.E.); adeboye05@yahoo.co.uk (A.A.A.); 2 Department of Histology and Cell Biology, Faculty of Medicine, Zagazig University, Zagazig 44519, Egypt; 3 Department of Health Promotion and Behavioral Sciences, University of Texas Health Sciences at Houston, Houston, TX 77030, USA; 4 Department of Management, Policy and Community Health, University of Texas Health Sciences at Houston, Houston, TX 77030, USA; rafeeky8@gmail.com; 5 Department of Internal Medicine, College of Medicine, University of Ha’il, Ha’il 2240, Saudi Arabia; mo.ghonim@uoh.edu.sa; 6 Department of Internal Medicine, Faculty of Medicine, Zagazig University, Zagazig 44519, Egypt

**Keywords:** SARS-CoV-2, COVID-19, lactoferrin, adjunctive, Egyptian COVID-19 management protocol

## Abstract

*Background and Objectives*: Preventive, adjunctive and curative properties of lactoferrin have been evaluated since the first wave of severe acute respiratory syndrome coronavirus (SARS-CoV), a viral respiratory disease, emerged 18 years ago. Despite the discovery of new vaccine candidates, there is currently no widely approved treatment for SARS-CoV-2 (COVID-19). Strict adherence to infection prevention and control procedures, as well as vaccines, can, however, prevent the spread of SARS-CoV-2. This study aimed to evaluate the efficacy of lactoferrin treatment in improving clinical symptoms and laboratory indices among individuals with mild to moderate coronavirus disease-19 (COVID-19). *Materials and Method*: A randomized, prospective, interventional pilot study conducted between 8 July and 18 September 2020 used a hospital-based sample of 54 laboratory-confirmed participants with mild to moderate symptoms of COVID-19. Randomization into a control and two treatment groups ensured all groups received the approved Egyptian COVID-19 management protocol; only treatment group participants received lactoferrin at different doses for seven days. Clinical symptoms and laboratory indices were assessed on Days 0, 2 and 7 after starting treatments. Mean values with standard deviation and one-way analysis of variance with least significant difference of demographic and laboratory data between control and treatment groups were calculated. *Results*: Our study showed no statistically significant difference among studied groups regarding recovery of symptoms or laboratory improvement. *Conclusions*: Further research into therapeutic properties particularly related to dosage, duration and follow-up after treatment with lactoferrin in individuals with COVID-19 is required.

## 1. Introduction

The novel severe acute respiratory syndrome coronavirus (SARS-CoV-2), the causative agent of coronavirus disease 2019 (COVID-19), was first discovered in Wuhan, China in December 2019. It causes a viral pneumonia which may progress to acute respiratory distress and death [1]. It was declared a pandemic by the World Health Organization on 11 February 2020 [2]. Currently, in spite of the development of new vaccine candidates, there is no universally accepted treatment for COVID-19. However, strict compliance with infection prevention and control protocols and vaccinations can stem the transmission of SARS-CoV-2 [2]. As a result, finding and developing preventative, adjuvant and curative treatments to treat COVID-19 is critical [3,4,5].

There is a wide scope of naturally occurring substances, for example, lactoferrin (Lf), that might be formed into expected drugs for treating COVID-19 [6,7]. Lactoferrin is a component of the innate immune system found in mucosal secretions of humans and animals [8]. It has the potential to treat a wide range of pathogens, including bacteria, protozoa, fungi and a wide range of naked and enveloped viruses [9]. Lf is a non-heme iron-binding globular glycoprotein with around 700 amino acids that belongs to the transferrin family and is the most often tested animal protein [10], with remarkable biocompatibility and safety profiles [11,12].

Lf has been shown to have a wide range of beneficial characteristics. Lactoferrin is found in substantial concentrations in neutrophils and serves as a preventive and pathogen-reduction agent in vitro and in vivo [13,14,15]. Lactoferrin deficiency may make some people more vulnerable to infection [16,17]. As a result, it is regarded as a crucial initial line of defense [8,18].

Lactoferrin has been found to have an anti-inflammatory effect on humans [19]. Furthermore, Lf chelates iron (Fe), rendering the ionized form of Fe ineffective against invading microbes [20]. Lf’s pathogen-reducing properties may include “direct” antiviral activities that have yet to be investigated thoroughly [7,21,22]. While there are some studies that have looked at the treatment effect of Lf on clinical symptoms and there are plenty that have focused on the overall impact of Lf [12,23], there is a dearth of studies that have explored the treatment effect of Lf on laboratory indices only and both clinical symptoms and laboratory indices simultaneously among individuals with SARS-CoV-2 infection [24]. Thus, the aim of this study was to evaluate the efficacy of lactoferrin treatment in improving clinical symptoms and laboratory indices in individuals with mild to moderate COVID-19. To the best of our knowledge, this is the first study to examine the efficacy of lactoferrin in the treatment of mild to moderate SARS-CoV-2 infection in North Africa.

## 2. Materials and Methods

This is a randomized, prospective, interventional pilot study using a hospital-based sample of 54 laboratory-confirmed (SARS-CoV-2 positive) participants with mild to moderate symptoms of COVID-19. Recruitment was carried out in the Department of Medicine at Al-Ahrar Hospital, Zagazig, Egypt from 8 July to 18 September 2020. Diagnosis was confirmed by a positive result of reverse transcriptase polymerase detection from a nasopharyngeal swab as indicated in the WHO guidelines [25].

The definition of mild to moderate COVID-19 was based on interim guidance from the WHO, 2020 [26]. COVID-19 has a wide clinical range, spanning from asymptomatic to mild, moderate, severe and critical disease. *Asymptomatic infection* is defined as patients who test positive for SARS-CoV-2 on a virologic test but do not develop COVID-19 symptoms, *mild illness* is defined as individuals who have COVID-19 symptoms but no evidence of viral pneumonia or hypoxia, *moderate illness* is defined as people who show clinical symptoms of pneumonia but not severe pneumonia, such as a SpO2 of less than 90% on room air, *severe illness* is defined as people who express clinical symptoms of pneumonia as well as one or more of the following: a respiratory rate of more than 30 breaths per minute, severe respiratory distress, or a SpO2 of less than 90% on room air and *critical illness* is defined as individuals who have acute respiratory distress syndrome (ARDS), septic shock and/or multiple organ dysfunction.

Eligibility criteria for inclusion in this study were participants over 20 years of age, positive for nasopharyngeal swab reverse transcriptase polymerase chain reaction (RT-PCR) for COVID-19 and blood oxygen saturation (SpO_2_) >93%. Exclusion criteria included pregnant and breastfeeding women, individuals confirmed to be allergic to milk protein, those with a medical history of bronchial hyperactivity or pre-existing respiratory diseases and ICU inpatients with COVID-19.

To maintain and determine the rights of patients without imperiling their protection and secrecy, informed verbal consent, instead of written consent, was obtained straightforwardly from all patients or their family members after providing a detailed disclosure of the research. The ability to get written informed consent was not achievable because of the inherent risky and contagious nature of the COVID-19 epidemic, the severe limitations put on regular visiting patients by hospital management and the necessity of gathering the information. Afterwards, the electronic medical records of all eligible participants were obtained and data related to medical history, medication history, laboratory diagnosis and non-contrast chest (CT) scans were gathered. In addition, all eligible participants underwent further laboratory tests comprising: complete blood count, liver and kidney function chemistry panels, coagulation profile, C-reactive protein, ferritin and lactate dehydrogenase (LDH) serum levels.

The blood samples of all eligible COVID-19-positive participants, body temperature and reported signs and symptoms were collected at Day 0, Day 2 and Day 7. Day 0 represents the first day of starting treatment and blood collection. Day 2 represents the second day of collecting samples after 48 h of starting treatment. Day 7 represents the third day of collecting samples after seven days of starting treatment.

Although the recruitment of eligible participants into the study was implemented using a convenience sampling method with a tendency to introduce various ingroup differences, this was minimized by randomization of participants into control group and treatment groups (Group I and II). The randomization process was double blinded wherein the investigators and study participants were not aware of individuals assigned to either the control or treatment groups.

According to Egypt’s approved COVID-19 patient management protocol [27], all eligible participants received care including: (a) intranasal oxygen to achieve oxygen saturation (SaO_2_) ≥90%; (b) oral hydroxychloroquine 400 mg two times a day during the first day and 200 mg twice daily for the next 6 days; (c) oral vitamin C 1 g daily; (d) oral zinc 600 mcg daily; (e) oral acetylcysteine 200 mg three times daily.

The eligible participants receiving standard protocol treatment were randomly assigned (as described above) into three groups. The first group was the Control Group; participants in the second group were designated as Group I; and participants in the third group were designated as Group II. Each group was made up of 18 participants. None of the participants in the Control Group received lactoferrin. Each participant in Group I received 200 mg lactoferrin orally once daily. Each participant in Group II received 200 mg lactoferrin orally twice daily. Those patients received lactoferrin sachets (Pravotin sachets produced by Hygint Pharmaceutical Innovation, Egypt). Each sachet contained lactoferrin 100 mg and was prepared by dissolving in half a glass of water or juice before meals [28]. The rationale behind giving each participant 200 mg lactoferrin twice daily was based on the recommended dosage documented on the technical card of drug. Each group was monitored using the Egyptian Health Ministry’s protocol.

### Statistical Analyses

Mean values with standard deviation (SD) of demographic and laboratory data were calculated. Mean was found to accurately represent the center distribution of our data after a test of normality was carried out. In addition, the proportion of each clinical characteristic was reported. For non-parametric data (clinical characteristics of participants), the chi-square test (significance level: *p* < 0.05) was used. To determine significant differences for parametric data between Control Group and treatment groups (Group I and II), one-way analysis of variance (ANOVA) was performed. Given the small but equal sample sizes within the control and treatment groups, ANOVA was the preferred test of choice since it is still robust for deviations from normality [29]. Significance level was set at a *p* value of less than 5% (*p* < 0.05) and 95% confidence interval levels were reported. All analyses were carried out using Statistical Package for the Social Sciences version 26 (SPSS v.26) and Graphpad Prism 6.

## 3. Results

Our study included 54 participants who were classified into three groups. The proportions of females in the Control group, Group I and Group II were 55.6%, 27.8% and 38.9%, respectively. Table 1 shows the demographic and clinical characteristics of the study participants. The mean ±SD ages (years) of the participants were: Control Group 48.93 ± 9.4, Group I 49.07 ± 8.5 and Group II 47.87 ± 11.3. There was no statistically significant difference in symptoms between participants in each group on Days 0, 2 and 7 (Table 1).

**Figure 1:**
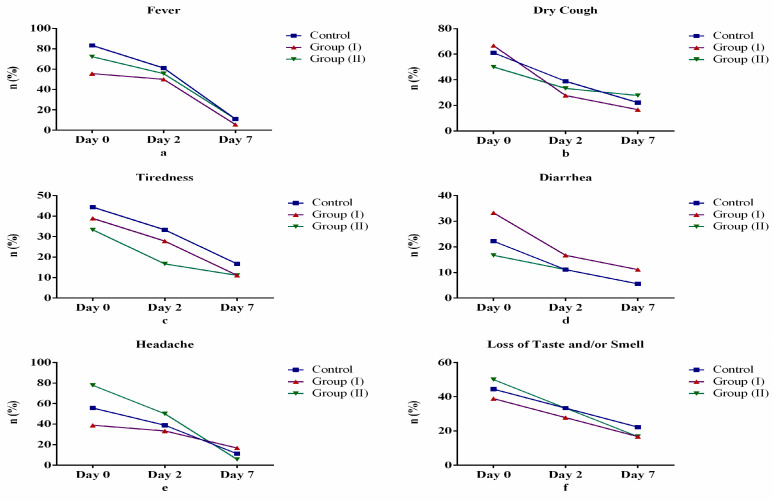
Comparison of clinical symptoms between control and treatment groups: (**a**) fever, (**b**) Dry cough, (**c**) tiredness, (**d**) diarrhea, (**e**) headache, (**f**) loss of sense of taste and/or smell.

In Table 1, we show a significant decrease in the number of participants reporting clinical symptoms (fever, dry cough, diarrhea, headache, loss of sense of taste and/or smell and tiredness) between Days 0, 2 and 7 across groups (Control Group, Group I and Group II). A similar significant decrease was observed across each group but with different proportionality. This observed decrease across groups was, however, not statistically significant (*p* > 0.05). In Figure 1, we show that study participants revealed overall improvement in symptoms of fever, dry cough, diarrhea, tiredness, headache and sense of smell and taste. Specifically, after the seventh day (Day 7) of starting treatment, fever (Figure 1a), diarrhea (Figure 1d) and headache (Figure 1e) revealed the greatest rate of improvement among Group II participants as expected when compared with other days after starting treatment and other groups. Conversely, dry cough (Figure 1b), tiredness (Figure 1c) and loss of taste and smell (Figure 1f) did not show expected rate of improvement among study participants in Group II.

**Figure 2:**
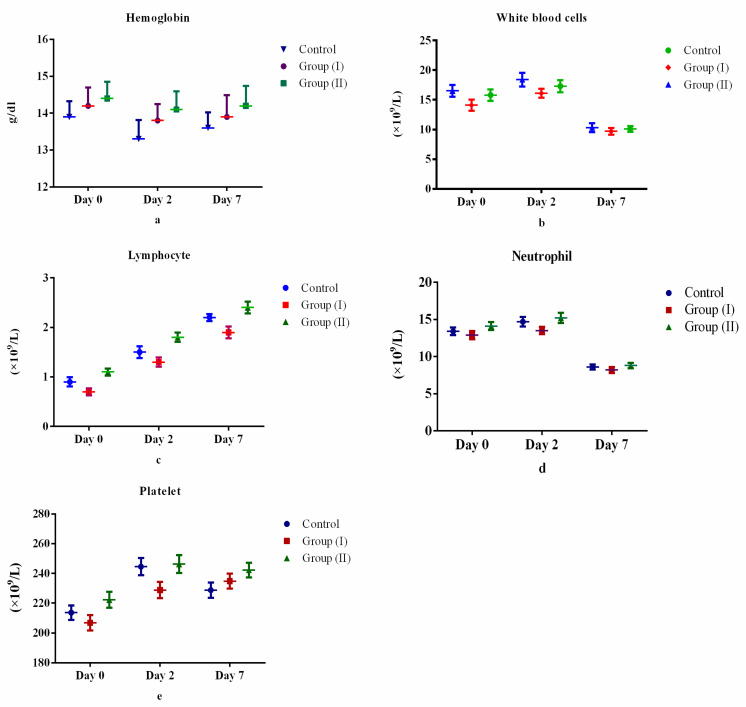
Comparison of complete blood count between control and treatment groups: (**a**) hemoglobin, (**b**) total white cell count, (**c**) lymphocytes, (**d**) neutrophils, (**e**) platelets.

**Figure 3:**
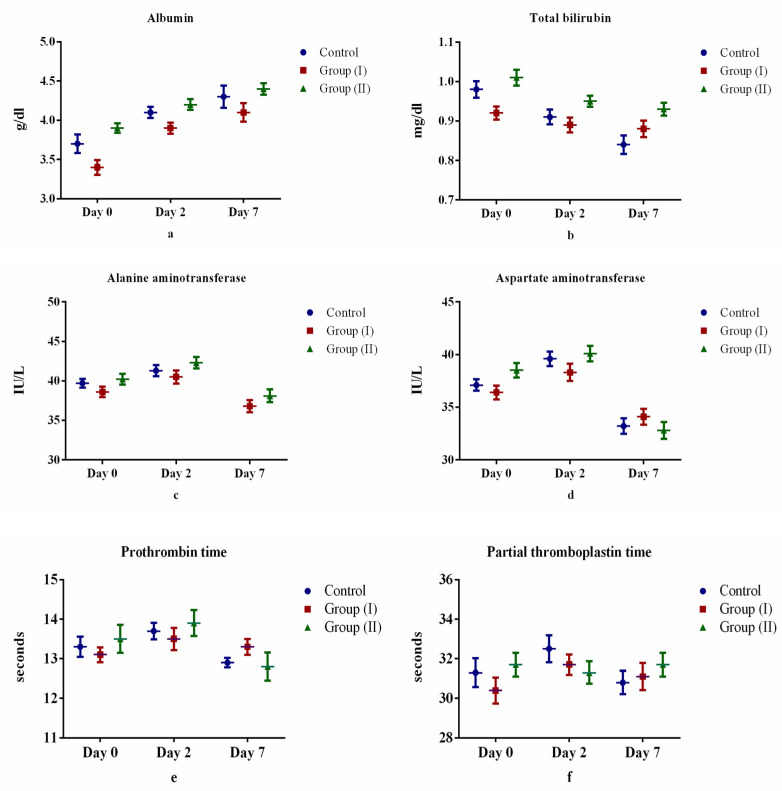
Comparison of liver function tests between control and treatment groups: (**a**) albumin, (**b**) total bilirubin, (**c**) alanine aminotransferase, (**d**) aspartate aminotransferase, (**e**) prothrombin time, (**f**) partial thromboplastin time.

**Figure 4:**
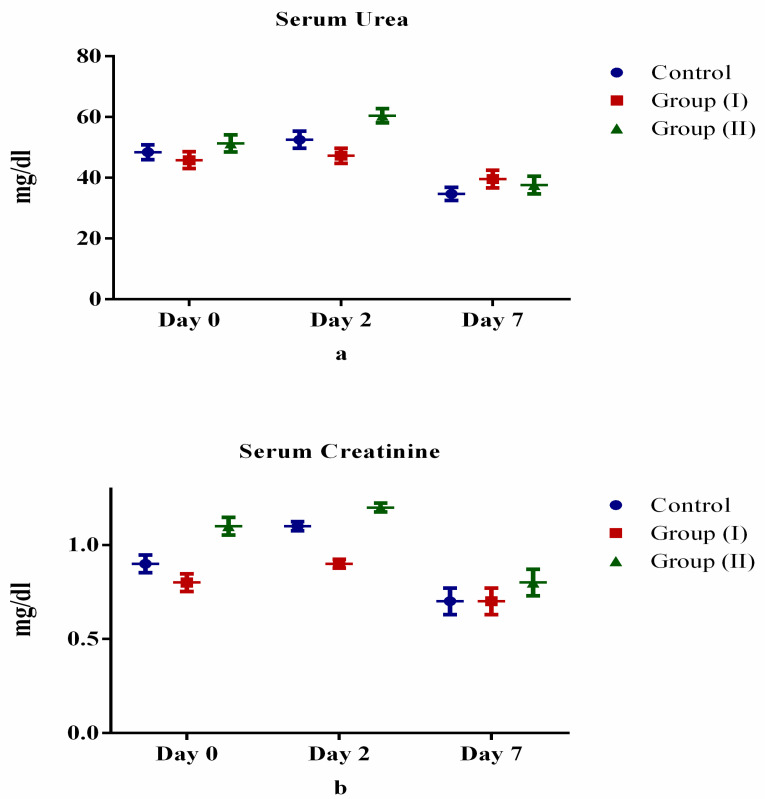
Comparison of kidney function tests between control and treatment groups: (**a**) urea, (**b**) creatinine.

**Figure 5:**
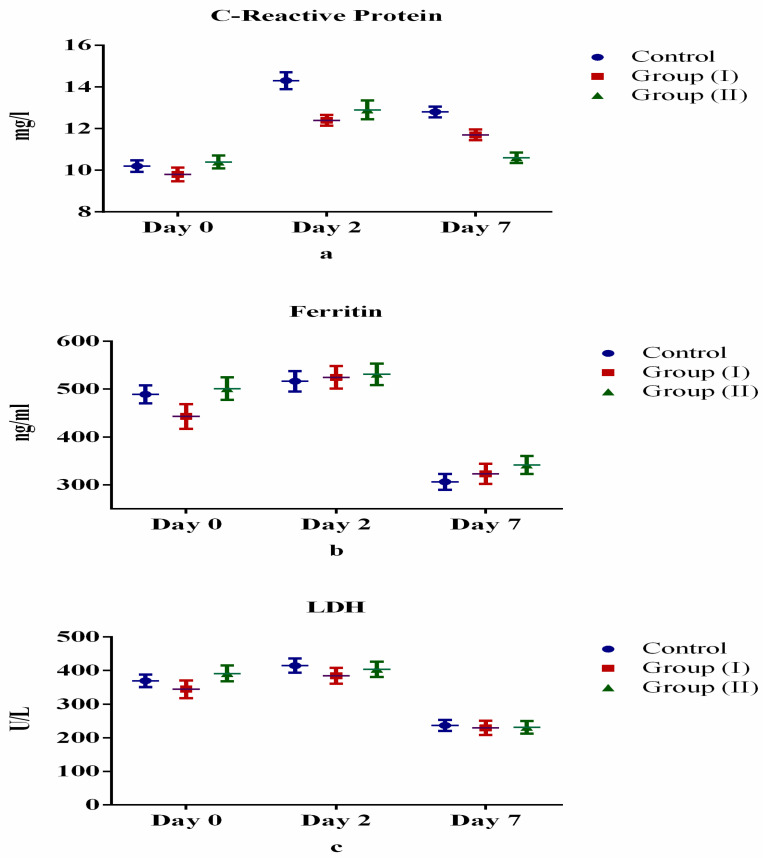
Comparison of acute inflammatory markers between control and treatment groups: (**a**) C-reactive protein, (**b**) ferritin, (**c**) lactate dehydrogenase.

Table 2 and Figure 2, Figure 3, Figure 4 and Figure 5 show the differences (both positive and negative trends) between the laboratory indices in the Control Group and treatment Group II (serum hemoglobin, total white cell count, lymphocytes, neutrophils, platelets, albumin, total bilirubin, alanine aminotransferase, aspartate aminotransferase, prothrombin time, partial thromboplastin time, serum urea, serum creatinine, C-reactive protein, serum ferritin and lactate dehydrogenase). These differences were particularly obvious on the seventh day (Day 7) after starting treatment. For example, lymphocyte count increased by 6.0% and 4.4% for Days 2 and 7 after starting treatment. Conversely, the observed differences between Control Group and treatment Group I were equivocal. However, none of these differences were statistically significant (*p* > 0.05).

## 4. Discussion

A pilot study of 54 symptomatic participants diagnosed with COVID-19 in Egypt was conducted. Although clinical and laboratory differences were observed between the control and treatment groups, these differences were not statistically significant.

Our study showed improvements in COVID-19 symptoms including fever, dry cough, diarrhea, headache, loss of sense of taste and/or smell and tiredness after the seventh day of starting treatment with Lf in all groups, however these changes showed no statistically significant difference among studied groups. These findings can be explained by the established broad-spectrum antiviral, anti-inflammatory and immunomodulatory properties of Lf [11,22,30,31,32,33].

In SARS-CoV-2 infection, serum hemoglobin levels, lymphocyte count and platelet count are expected to decline [34,35,36], while total white cell count and neutrophil count are expected to rise [35]. Accordingly, as expected, treatment of study participants with twice daily doses of Lf (200 mg orally twice daily) revealed increases in serum hemoglobin level, lymphocyte count and in platelet count between control and treatment groups through treatment days. This expected laboratory indices finding is similar to prior studies [37,38]. The plausible explanation for this observation can be attributed to broad spectrum anti-inflammatory and anti-infective properties of Lf. An interesting result in our study was the increase in both white blood cell and neutrophil counts between control and treatment groups from Day 0 to Day 2 followed by decreases in these counts in Day 7 as compared to Day 2. A logical explanation for these unanticipated findings may be that a higher dose and longer duration of treatment with Lf and follow-up of participants is required to observe the expected effect of decrease in both total white cell and neutrophil counts.

In previous studies, liver function tests in individuals with COVID-19 revealed mixed findings: serum albumin levels are reduced; total bilirubin, alanine aminotransferase and aspartate aminotransferase levels are elevated while prothrombin time and partial thromboplastin time are shortened [39,40]. In our study, following treatment with twice daily doses of Lf, serum albumin level increased as expected between control and treatment groups. All other liver function test indices were equivocal with Lf treatment; perhaps a higher dose and longer duration of treatment and follow-up after treatment with Lf are required to observe the expected effects.

Prior research indicated elevation of serum urea and creatinine levels in individuals with SARS-CoV-2 infection [41,42]. However, treatment with Lf has been shown to be renoprotective due to its antioxidant and other therapeutic properties [43]. Findings in our study were contrary to expected results, perhaps due to variation in dosage, duration and follow-up after treatment administered to the participants.

C-reactive protein, serum ferritin and lactate dehydrogenase levels in persons with COVID-19 were shown in previous studies to be elevated [44,45,46]. The levels of C-reactive protein after treatment with Lf in our study declined as expected between control and treatment groups. This could be an indication of the broad-spectrum anti-inflammatory property of Lf. Nevertheless, the elevated serum ferritin and lactate dehydrogenase levels observed in our study are contrary to the therapeutic effect of Lf. This may be due to the differences in dosage, duration and follow-up of the participants after Lf treatment.

This pilot study in not without inherent limitations. Firstly, the limited sample size of hospital-based participants potentially reduced the power to observe expected statistically significant findings. However, we attempted to mitigate this limitation by making extensive use of descriptive statistics and allocating resources to maximize inference for the most important parameters. Secondly, the combination of short duration of treatment, relative lower dose and lack of follow-up after treatment with Lf administered to participants may have potentially impacted the expected results.

## 5. Conclusions

The preventive, adjunctive and curative properties of lactoferrin have been evaluated since the emergence of the first wave of the severe acute respiratory syndrome (SARS), a viral respiratory disease, 18 years ago. LF supplementation is undoubtedly a promising field for additional research, however the results of this study do not allow for a definitive conclusion about its potential benefits as a support therapy. Further studies with larger samples, as well as longer-term trials to evaluate if viral clearance can be sustained with continuous administration of Lf, are needed to better understand the role of Lf in treating SARS-CoV-2.

## Figures and Tables

**Table 1:** Demographic and clinical characteristics of participants.

Variables	Control Group	Group I	Group II	*p*-Value
**Age** (years) (mean ± SD)	48.93 ± 9.4	49.07 ± 8.5	47.87 ± 11.3	***-***
**Female** Sex, *n* (%)	10 (55.6%)	5 (27.8%)	7 (38.9%)	***-***
**Male** Sex, *n* (%)	8 (44.4%)	13 (72.2%)	11 (61.1%)	***-***
**Fever**				
Day 0	15 (83.3%)	10 (55.6%)	13 (72.2%)	0.185
Day 2	11 (61.1%)	9 (50.0%)	10 (55.6%)	0.799
Day 7	2 (11.1%)	1 (5.6%)	2 (11.1%)	0.802
**Dry Cough**				
Day 0	11 (61.1%)	12 (66.7%)	9 (50.0%)	0.585
Day 2	7 (38.9%)	5 (27.8%)	6 (33.3%)	0.779
Day 7	4 (22.2%)	3 (16.7%)	5 (27.8%)	0.725
**Tiredness**				
Day 0	8 (44.4%)	7 (38.9%)	6 (33.3%)	0.792
Day 2	6 (33.3%)	5 (27.8%)	3 (16.7%)	0.509
Day 7	3 (16.7%)	2 (11.1%)	2 (11.1%)	0.849
**Diarrhea**				
Day 0	4 (22.2%)	6 (33.3%)	3 (16.7%)	0.492
Day 2	2 (11.1%)	3 (16.7%)	2 (11.1%)	0.849
Day 7	1 (5.6%)	2 (11.1%)	1 (5.6%)	0.763
**Headache**				
Day 0	10 (55.6%)	12 (38.9%)	14 (77.8%)	0.368
Day 2	7 (38.9%)	6 (33.3%)	9 (50.0%)	0.585
Day 7	2 (11.1%)	3 (16.7%)	1 (5.6%)	0.570
**Loss of Taste and/or Smell**				
Day 0	8 (44.4%)	7 (38.9%)	9 (50.0%)	0.799
Day 2	6 (33.3%)	5 (27.8%)	6 (33.3%)	0.918
Day 7	4 (22.2%)	3 (16.7%)	3 (16.7%)	0.885

SD = standard deviation; *n* = number.

**Table 2:** Laboratory characteristics of participants (mean ± standard deviation).

Variables	Control Group	Group I	Group II	*p*-Value
**Hemoglobin (g/dL)**				
Day 0	13.9 ± 2.2	14.2 ± 1.9	14.4 ± 2.1	0.797
Day 2	13.3 ± 1.8	13.8 ± 2.5	14.1 ± 1.9	0.474
Day 7	13.6 ± 1.8	13.9 ± 2.1	14.2 ± 2.3	0.657
**White blood cells (×10^9^/L)**				
Day 0	15.8 ± 4.1	14.1 ± 3.9	16.5 ± 4.2	0.215
Day 2	17.3 ± 4.3	16.1 ± 3.2	18.4 ± 4.8	0.254
Day 7	10.1 ± 2.1	9.7 ± 2.5	10.3 ± 3.2	0.786
**Lymphocyte (×10^9^/L)**				
Day 0	0.9 ± 0.4	0.7 ± 0.3	1.1 ± 0.3	0.005
Day 2	1.5 ± 0.5	1.3 ± 0.4	1.8 ± 0.4	0.003
Day 7	2.2 ± 0.3	1.9 ± 0.5	2.4 ± 0.5	0.002
**Neutrophil (×10^9^/L)**				
Day 0	13.4 ± 2.2	12.9 ± 2.4	14.1 ± 2.3	0.311
Day 2	14.7 ± 2.7	13.5 ± 2.1	15.2 ± 2.9	0.139
Day 7	8.6 ± 1.5	8.2 ± 1.7	8.8 ± 1.5	0.58
**Platelet (×10^9^/L)**				
Day 0	213.6 ± 20.5	206.8 ± 21.5	222.2 ± 99.7	0.744
Day 2	244.5 ± 24.5	234.5 ± 21.1	246.2 ± 99.5	0.822
Day 7	228.7 ± 21.5	216.3 ± 23.4	242.2 ± 91.8	0.392
**Albumin (g/dL)**				
Day 0	3.7 ± 0.5	3.4 ± 0.4	3.9 ± 0.2	0.006
Day 2	4.1 ± 0.3	3.9 ± 0.3	4.2 ± 0.3	0.014
Day 7	4.3 ± 0.6	4.1 ± 0.5	4.4 ± 0.3	0.22
**Total bilirubin (mg/dL)**				
Day 0	0.98 ± 0.1	0.92 ± 04	1.01 ± 0.2	0.559
Day 2	0.91 ± 0.1	0.89 ± 0.1	0.95 ± 0.3	0.592
Day 7	0.84 ± 0.2	0.88 ± 0.3	0.93 ± 0.1	0.334
**Alanine aminotransferase (IU/L)**				
Day 0	39.7 ± 2.3	38.6 ± 2.8	40.2 ± 2.9	0.163
Day 2	41.3 ± 2.9	40.5 ± 3.5	42.3 ± 3.1	0.208
Day 7	37.5 ± 3.1	36.8 ± 3.2	38.1 ± 3.4	0.454
**Aspartate aminotransferase (IU/L)**				
Day 0	37.1 ± 2.9	36.4 ± 3.5	38.5 ± 3.1	0.139
Day 2	39.6 ± 2.3	38.3 ± 2.8	40.1 ± 2.9	0.103
Day 7	33.2 ± 3.1	34.1 ± 3.2	32.8 ± 3.4	0.456
**Prothrombin time (seconds)**				
Day 0	13.3 ± 1.1	13.1 ± 0.8	13.5 ± 1.5	0.536
Day 2	13.7 ± 0.9	13.5 ± 1.2	13.9 ± 1.4	0.484
Day 7	12.9 ± 0.5	13.3 ± 0.9	12.8 ± 1.5	0.044
**Partial thromboplastin time (seconds)**				
Day 0	31.3 ± 3.1	30.4 ± 2.8	31.3 ± 2.4	0.577
Day 2	32.5 ± 2.9	31.7 ± 2.2	31.7 ± 2.5	0.575
Day 7	30.8 ± 2.5	31.1 ± 2.9	29.7 ± 2.3	0.23
**Serum urea (mg/dL)**				
Day 0	48.4 ± 10.3	45.8 ± 11.8	51.3 ± 11.9	0.243
Day 2	52.5 ± 11.9	47.2 ± 10.5	60.4 ± 10.1	0.002
Day 7	34.7 ± 9.1	39.6 ± 12.2	37.6 ± 12.4	0.444
**Serum creatinine (mg/dL)**				
Day 0	0.9 ± 0.2	0.8 ± 0.2	1.1 ± 0.2	0.000
Day 2	1.1 ± 0.1	0.9 ± 0.1	1.2 ± 0.1	0.000
Day 7	0.7 ± 0.3	0.7 ± 0.3	0.8 ± 0.3	0.349
**C-reactive protein (mg/L)**				
Day 0	10.2 ± 1.2	9.8 ± 1.4	10.4 ± 1.3	0.368
Day 2	14.3 ± 1.7	12.4 ± 1.1	12.9 ± 1.9	0.003
Day 7	12.8 ± 1.1	11.7 ± 1.1	10.6 ± 1.1	0.000
**Ferritin (ng/mL)**				
Day 0	488.9 ± 83.6	443.1 ± 110.3	501.1 ± 101.8	0.195
Day 2	516.3 ± 90.2	524.6 ± 100.6	531 ± 95.1	0.898
Day 7	306.3 ± 71.6	323 ± 90.3	341.5 ± 80.7	0.436
**Lactate dehydrogenase (U/L)**				
Day 0	369.3 ± 81.2	344.6 ± 110.8	391.2 ± 100.4	0.382
Day 2	414.4 ± 90.1	384.2 ± 100.6	403.5 ± 95.9	0.647
Day 7	236.8 ± 70.3	229.5 ± 90.4	231.4 ± 80.7	0.961

## Data Availability

The datasets generated and/or analyzed during the current study are available from the corresponding author upon request.

## Abbreviations

SARS-CoV-2: severe acute respiratory syndrome coronavirus-2; COVID-19: Coronavirus disease; Lf: Lactoferrin; Fe: Iron; WHO: World Health Organization; RT-PCR: reverse transcriptase polymerase chain reaction; LDH: Lactate dehydrogenase; ACE2: Angiotensin-converting enzyme 2; HSPGs: Heparan sulfate proteoglycans; IL: Interleukin; TNF alpha: Tumor Necrosis Factor alpha; CRP: C-reactive protein.

## References

[1] Dhama K., Sharun K., Tiwari R., Sircar S., Bhat S., Malik Y.S., Singh K.P., Chaicumpa W., Bonilla-Aldana D.K., Rodriguez-Morales A.J. (2020). Coronavirus Disease 2019—COVID-19. Clin. Microbiol. Rev..

[2] World Health Organization (2020). Coronavirus (COVID-19) Events as They Happen. https://www.who.int/emergencies/diseases/novelcoronavirus-2019/events-as-they-happen.

[3] Zhavoronkov A., Aladinskiy V., Zhebrak A., Zagribelnyy B., Terentiev V., Bezrukov D.S., Polykovskiy D., Shayakhmetov R., Filimonov A., Orekhov P. (2020). Potential COVID-2019 3C-Like Protease Inhibitors Designed Using Generative Deep Learning Approaches.

[4] Angeletti S., Benvenuto D., Bianchi M., Giovanetti M., Pascarella S., Ciccozzi M. (2020). COVID-2019: The role of the nsp2 and nsp3 in its pathogenesis. J. Med. Virol..

[5] Costagliola G., Spada E., Comberiati P., Peroni D.G. (2021). Could nutritional supplements act as therapeutic adjuvants in COVID-19?. Ital. J. Pediatr..

[6] Yang Y., Cao L., Gao H., Wu Y., Wang Y., Fang F., Rao Y. (2019). Discovery, optimization, and target identi-fication of novel potent broad-spectrum antiviral inhibitors. J. Med. Chem..

[7] Feng M., Fei S., Xia J., Labropoulou V., Swevers L., Sun J. (2020). Antimicrobial Peptides as Potential Antiviral Factors in Insect Antiviral Immune Response. Front. Immunol..

[8] Sienkiewicz M., Jaśkiewicz A., Tarasiuk A., Fichna J. (2021). Lactoferrin: An overview of its main functions, immunomodulatory and antimicrobial role, and clinical significance. Crit. Rev. Food Sci. Nutr..

[9] Buey B., Bellés A., Latorre E., Abad I., Pérez M.D., Grasa L., Sánchez L. (2021). Comparative effect of bovine buttermilk, whey, and lactoferrin on the innate immunity receptors and oxidative status of intestinal epithelial cells. Biochem. Cell Biol..

[10] Wang B., Timilsena Y.P., Blanch E., Adhikari B. (2019). Lactoferrin: Structure, function, denaturation and digestion. Crit. Rev. Food Sci. Nutr..

[11] Wang Y., Wang P., Wang H., Luo Y., Wan L., Jiang M., Chu Y. (2020). Lactoferrin for the treatment of COVID-19. Exp. Ther. Med..

[12] Serrano G., Kochergina I., Albors A., Diaz E., Oroval M., Hueso G., Serrano J.M. (2020). Liposomal Lactoferrin as Potential Preventative and Cure for COVID-19. Int. J. Res. Health Sci..

[13] Azhar J., Mohammadabadi T., Babar M.E., Hussain T. (2020). Milk lactoferrin: A probable immunological agent against sars-cov-2: A review. Basrah J. Agric. Sci..

[14] Hao L., Shan Q., Wei J., Ma F., Sun P. (2019). Lactoferrin: Major Physiological Functions and Applications. Curr. Protein Pept. Sci..

[15] Khajeh E., Jamshidian-Mojaver M., Naeemipour M., Farzin H. (2021). The Identification of a Novel Peptide Derived from Lactoferrin Isolated from Camel Milk with Potential Antimicrobial Activity. Iran. J. Med. Microbiol..

[16] Kell D.B., Heyden E.L., Pretorius E. (2020). The biology of lactoferrin, an iron-binding protein that can help de-fend against viruses and bacteria. Front. Immunol..

[17] Quintieri L., Caputo L., Monaci L., Cavalluzzi M.M., Denora N. (2020). Lactoferrin-derived peptides as a con-trol strategy against skinborne staphylococcal biofilms. Biomedicines.

[18] Farid A., El Shemy M.A., Nafie E., Hegazy A.M., Abdelhiee E.Y. (2021). Anti-inflammatory, anti-oxidant and hepatoprotective effects of lactoferrin in rats. Drug Chem. Toxicol..

[19] Shahidi F., Roshanak S., Javadmanesh A., Yazdi F.T., Pirkhezranian Z., Azghandi M. (2020). Evaluation of antimicrobial properties of bovine lactoferrin against foodborne pathogenic microorganisms in planktonic and bio-film forms (in vitro). J. Consum. Prot. Food Saf..

[20] Padrão J., Ribeiro S., Lanceros-Méndez S., Rodrigues L.R., Dourado F. (2020). Effect of bacterial nanocellulose binding on the bactericidal activity of bovine lactoferrin. Heliyon.

[21] Lodhi A.M., Aslam P., Sajid K., Zulfiqar K. (2019). Lactoferrin as Nutraceutical Protein from Milk. J. Nutraceuticals Food Sci..

[22] Peroni D.G. (2020). Viral infections: Lactoferrin, a further arrow in the quiver of prevention. J. Pediat. Neonatal Individ. Med. (JPNIM).

[23] Hu Y., Meng X., Zhang F., Xiang Y., Wang J. (2021). The in vitro antiviral activity of lactoferrin against common human coronaviruses and SARS-CoV-2 is mediated by targeting the heparan sulfate co-receptor. Emerg. Microbes Infect..

[24] Campione E., Lanna C., Cosio T., Rosa L., Conte M.P., Iacovelli F., Romeo A., Falconi M., del Vecchio C., Franchin E. (2020). Lactoferrin as Potential Supplementary Nutraceutical Agent in COVID-19 Patients: In vitro and in vivo Preliminary Evidences. BioRxiv.

[25] World Health Organization (2020). Laboratory Testing for 2019 Novel Coronavirus (2019-nCoV) in Suspected Human Cases: Interim Guidance, 14 January 2020.

[26] Xu Y.-H., Dong J.-H., An W.-M., Lv X.-Y., Yin X.-P., Zhang J.-Z., Dong L., Ma X., Zhang H.-J., Gao B.-L. (2020). Clinical and computed tomographic imaging features of novel coronavirus pneumonia caused by SARS-CoV-2. J. Infect..

[27] Sorour K., El-Menshawy H. (2020). A Proposed Protocol for the Management of COVID-19 in Egypt. AfricArXiv.

[28] Elmenam H.S., Farouk M.H. (2021). Bovine lactoferrin in preterm labor with sterile inflammation. Sci. J. Al-Azhar Med. Fac. Girls.

[29] Sullivan L.M., Weinberg J., Keaney J.F. (2016). Common statistical pitfalls in basic science research. J. Am. Heart Assoc..

[30] Chang R., Ng T.B., Sun W.-Z. (2020). Lactoferrin as potential preventative and adjunct treatment for COVID-19. Int. J. Antimicrob. Agents.

[31] Salaris C., Scarpa M., Elli M., Bertolini A., Guglielmetti S., Pregliasco F., Castagliuolo I. (2021). Protective effects of lactoferrin against SARS-CoV-2 infection in vitro. Nutrients.

[32] Niaz B., Saeed F., Ahmed A., Imran M., Maan A.A., Khan M.K.I., Tufail T., Anjum F.M., Hussain S., Suleria H.A.R. (2019). Lactoferrin (LF): A natural antimicrobial protein. Int. J. Food Prop..

[33] Kruzel M.L., Olszewska P., Pazdrak B., Krupinska A.M., Actor J.K. (2021). New insights into the systemic effects of oral lactoferrin: Transcriptome profiling. Biochem. Cell Biol..

[34] Cavezzi A., Troiani E., Corrao S. (2020). COVID-19: Hemoglobin, Iron, and Hypoxia beyond Inflammation. A Narrative Review. Clin. Pract..

[35] Anurag A., Jha P.K., Kumar A. (2020). Differential white blood cell count in the COVID-19: A cross-sectional study of 148 patients. Diabetes Metab. Syndr. Clin. Res. Rev..

[36] Wool G.D., Miller J.L. (2021). The Impact of COVID-19 Disease on Platelets and Coagulation. Pathobiology.

[37] Cutone A., Ianiro G., Lepanto M.S., Rosa L., Valenti P., Bonaccorsi di Patti M.C., Musci G. (2020). Lactoferrin in the Prevention and Treatment of Intestinal Inflammatory Pathologies Associated with Colorectal Cancer Development. Cancers.

[38] El-Khawaga A., Abdelmaksoud H. (2019). Effect of Lactoferrin Supplementation on Iron Deficiency Anemia in Primary School Children. Int. J. Med. Arts.

[39] Cai Q., Huang D., Yu H., Zhu Z., Xia Z., Su Y., Li Z., Zhou G., Gou J., Qu J. (2020). COVID-19: Abnormal liver function tests. J. Hepatol..

[40] Bertolini A., Van De Peppel I.P., Bodewes F.A., Moshage H., Fantin A., Farinati F., Fiorotto R., Jonker J.W., Strazzabosco M., Verkade H.J. (2020). Abnormal Liver Function Tests in Patients with COVID-19: Relevance and Potential Pathogenesis. Hepatology.

[41] Bajwa H., Riaz Y., Ammar M., Farooq S., Yousaf A. (2020). The Dilemma of Renal Involvement in COVID-19: A Systematic Review. Cureus.

[42] Liu Y.-M., Xie J., Chen M.-M., Zhang X., Cheng X., Li H., Zhou F., Qin J.-J., Lei F., Chen Z. (2021). Kidney Function Indicators Predict Adverse Outcomes of COVID-19. Med.

[43] Hsu Y.-H., Chiu I.-J., Lin Y.-F., Chen Y.-J., Lee Y.-H., Chiu H.-W. (2020). Lactoferrin Contributes a Renoprotective Effect in Acute Kidney Injury and Early Renal Fibrosis. Pharmaceutics.

[44] Wang G., Wu C., Zhang Q., Wu F., Yu B., Lv J., Li Y., Li T., Zhang S., Wu C. (2020). C-Reactive Protein Level May Predict the Risk of COVID-19 Aggravation. Open Forum Infect. Dis..

[45] Cheng L., Li H., Li L., Liu C., Yan S., Chen H., Li Y. (2020). Ferritin in the coronavirus disease 2019 (COVID-19): A systematic review and meta-analysis. J. Clin. Lab. Anal..

[46] Henry B.M., Aggarwal G., Wong J., Benoit S., Vikse J., Plebani M., Lippi G. (2020). Lactate dehydrogenase levels predict coronavirus disease 2019 (COVID-19) severity and mortality: A pooled analysis. Am. J. Emerg. Med..

